# Plasma glial fibrillary acidic protein is elevated in cognitively normal older adults at risk of Alzheimer’s disease

**DOI:** 10.1038/s41398-020-01137-1

**Published:** 2021-01-11

**Authors:** Pratishtha Chatterjee, Steve Pedrini, Erik Stoops, Kathryn Goozee, Victor L. Villemagne, Prita R. Asih, Inge M. W. Verberk, Preeti Dave, Kevin Taddei, Hamid R. Sohrabi, Henrik Zetterberg, Kaj Blennow, Charlotte E. Teunissen, Hugo M. Vanderstichele, Ralph N. Martins

**Affiliations:** 1 Department of Biomedical Sciences, Macquarie University, North Ryde, NSW Australia; 2 School of Medical and Health Sciences, Edith Cowan University, Joondalup, WA Australia; 3 ADx NeuroSciences, Gent, Belgium; 4 KaRa Institute of Neurological Diseases, Macquarie Park, NSW Australia; 5 Anglicare, Castle Hill Sydney, NSW Australia; 6 School of Psychiatry and Clinical Neurosciences, University of Western Australia, Crawley, WA Australia; 7 The Cooperative Research Centre for Mental Health, Carlton South, Australia; 8 Department of Molecular Imaging & Therapy, Austin Health, Melbourne, VIC Australia; 9 Neurochemistry Laboratory, Department of Clinical Chemistry, Amsterdam Neuroscience, Amsterdam University Medical Centers, Amsterdam, Netherlands; 10 Australian Alzheimer’s Research Foundation, Nedlands, WA Australia; 11 Centre for Healthy Ageing, School of Psychology and Exercise Science, College of Science, Health, Engineering and Education, Murdoch University, Murdoch, WA Australia; 12 Department of Psychiatry and Neurochemistry, Institute of Neuroscience and Physiology, University of Gothenburg, Mölndal, Sweden; 13 Clinical Neurochemistry Laboratory, Sahlgrenska University Hospital, Mölndal, Sweden; 14 Department of Neurodegenerative Disease, UCL Institute of Neurology, Queen Square, London, United Kingdom; 15 UK Dementia Research Institute at UCL, London, UK; 16 Biomarkable, Gent, Belgium

**Keywords:** Molecular neuroscience, Diagnostic markers

## Abstract

Glial fibrillary acidic protein (GFAP), an astrocytic cytoskeletal protein, can be measured in blood samples, and has been associated with Alzheimer’s disease (AD). However, plasma GFAP has not been investigated in cognitively normal older adults at risk of AD, based on brain amyloid-β (Aβ) load. Cross-sectional analyses were carried out for plasma GFAP and plasma Aβ1–42/Aβ1–40 ratio, a blood-based marker associated with brain Aβ load, in participants (65–90 years) categorised into low (Aβ−, *n* = 63) and high (Aβ+, *n* = 33) brain Aβ load groups via Aβ positron emission tomography. Plasma GFAP, Aβ1–42, and Aβ1–40 were measured using the Single molecule array (Simoa) platform. Plasma GFAP levels were significantly higher (*p* < 0.00001), and plasma Aβ1–42/Aβ1–40 ratios were significantly lower (*p* < 0.005), in Aβ+ participants compared to Aβ− participants, adjusted for covariates age, sex, and apolipoprotein E-ε4 carriage. A receiver operating characteristic curve based on a logistic regression of the same covariates, the base model, distinguished Aβ+ from Aβ− (area under the curve, AUC = 0.78), but was outperformed when plasma GFAP was added to the base model (AUC = 0.91) and further improved with plasma Aβ1–42/Aβ1–40 ratio (AUC = 0.92). The current findings demonstrate that plasma GFAP levels are elevated in cognitively normal older adults at risk of AD. These observations suggest that astrocytic damage or activation begins from the pre-symptomatic stage of AD and is associated with brain Aβ load. Observations from the present study highlight the potential of plasma GFAP to contribute to a diagnostic blood biomarker panel (along with plasma Aβ1–42/Aβ1–40 ratios) for cognitively normal older adults at risk of AD.

## Introduction

Alzheimer’s disease (AD) is the most common form of dementia and it is estimated that globally over 50 million people are living with AD or other forms of dementia^1^. Currently, there is no cure or effective treatment for AD despite all scientific efforts and therefore, more recent clinical trials are focussing on prevention programmes for AD, thereby requiring the identification of populations at risk of AD.

Extracellular amyloid-β (Aβ) plaques and intracellular neurofibrillary tangles comprising tau are the major neuropathological hallmarks of AD and while a post-mortem examination identifying these hallmarks is relied upon for a confirmative diagnosis, Aβ and tau neuropathology associated with AD can be identified in vivo via positron emission tomography (PET) and cerebrospinal fluid (CSF) analysis 15–20 years prior to symptom onset^2^. However, the cost of PET imaging, the throughput of imaging in general and the invasiveness of lumbar puncture, required for CSF sample collection, restrict the implementation of these markers in standard clinical practice and as screening tools in clinical trials. In contrast, the cost effective and less invasive nature of blood-based biomarkers could serve as attractive surrogate markers for initial clinical diagnostic testing and screening for clinical trials.

Glial fibrillary acidic protein (GFAP) is an astrocytic cytoskeletal protein that serves as a marker of abnormal activation and proliferation of astrocytes due to neuronal damage, also known as astrogliosis^3^. Astrogliosis has also been observed around Aβ plaques from the prodromal stages of AD, such as the mild cognitive impairment stage^4^, and GFAP expression has been reported to correlate with Aβ plaque density in AD brain tissue^5^. In addition, higher GFAP levels have been reported in CSF samples from individuals with AD and other dementias, compared to healthy controls^6^.

Interestingly, relatively recent studies have also reported higher GFAP levels in the blood in early and late-onset AD^7–9^. GFAP levels in the blood were also observed to inversely correlate with cognition^8^ and positively correlate with the extent of white matter injury^7^. The current study investigated whether elevated GFAP levels in the blood precede the onset of the clinical symptoms of AD in cognitively normal older adults at risk of AD.

Given that the onset of abnormal brain Aβ load build-up assessed using PET begins as early as two decades prior to the clinical manifestation of AD, and is a prodromal feature and biomarker of AD^2,10^, plasma GFAP levels were compared between cognitively normal older adults with low brain Aβ load (Aβ−) and cognitively normal older adults at risk of AD, due to high brain Aβ load, (Aβ+)^11^. Our hypothesis was that plasma GFAP levels will be higher in the Aβ+ group compared to the Aβ− group.

The current study also evaluated the potential of plasma GFAP in differentiating between Aβ+ and Aβ− individuals. In addition, given that the association between plasma Aβ42/Aβ40 ratios and brain Aβ load has been extensively reported^12–15^, this study also evaluated the combined potential of plasma GFAP and plasma Aβ1–42/Aβ1–40 ratios in discriminating between Aβ+ and Aβ− individuals. Furthermore, associations of plasma GFAP with the AD risk factors, cognitive performance, and neurodegeneration marker, neurofilament light (NF-L), were also assessed in the present study.

## Materials and methods

### Participants and cognitive assessments

Participants in the current study were from the Kerr Anglican Retirement Village Initiative in Aging Health (KARVIAH) cohort^16^. All participants from the KARVIAH cohort met the inclusion and exclusion criteria, wherein the inclusion criteria comprised an age range of 65–90 years, good general health, no known significant cerebral vascular disease, fluent in English, adequate/corrected vision and hearing to enable testing, and no dementia or other pathological cognitive impairment, as primarily screened by a Montreal Cognitive Assessment (MoCA) score ≥26. MoCA scores lying between 18 and 25 were assessed on a case by case basis by the study neuropsychologist following stratification of scores, using age and education-adjusted norms^17^. The exclusion criteria comprised, previous diagnosis of dementia^18^, presence of acute functional psychiatric disorder (including lifetime history of schizophrenia or bipolar disorder), history of stroke, severe or extremely severe depression (based on the Depression Anxiety Stress Scales; DASS), and uncontrolled hypertension (systolic BP > 170 mm Hg or diastolic BP > 100 mm Hg). From the volunteers who met the inclusion and exclusion criteria (*n* = 134), 105 participants underwent neuroimaging, neuropsychometric evaluation, and blood collection since the remaining participants declined undergoing neuroimaging or withdrew from the study. Within these 105 participants, 100 participants were considered to have normal global cognition based on their Mini-Mental State Examination (MMSE; scores can range from 0 to 30, with higher scores indicating better cognitive function)^19^ wherein, a cut-off score <26 was employed to screen out potential dementia patients. Plasma GFAP concentrations were measured in 96 of the 100 participants, and plasma Aβ1–40 and Aβ1–42 concentrations were measured in 95 of these 100 participants. However, the total set of GFAP, Aβ1–40, and Aβ1–42 concentrations were available in 94 of these participants. In addition, participants with a Memory Assessment Clinic-Questionnaire (MAC-Q) score of 25–35 were considered as subjective memory complainers (SMCs, *n* = 74; a specific form of subjective cognitive decline, defined by self-reported memory complaints), while those with a MAC-Q score ≤24 were considered as non-complainers (*n* = 22). Details of the participants included within the current study have been illustrated in Supplementary Fig. 1.

Further, cognitive measures were calculated for verbal and visual episodic memory, working memory and executive function, as well as for a global composite score, that included verbal and visual episodic memory, working memory and executive function and MMSE scores, for each participant as described previously^20^.

All volunteers provided written informed consent prior to participation, and the Bellberry Human Research Ethics Committee, Australia (reference number 2012-09-1086) and the Macquarie University Human Research Ethics Committee (reference number 5201701078) provided approval for the study.

### Evaluation of neocortical amyloid-β load via PET

All study participants were imaged within 3 months of blood collection wherein participants underwent Aβ PET imaging with ^18^F-florbetaben (FBB) at Macquarie Medical Imaging in Sydney, Australia. Participants were administered an intravenous bolus of FBB slowly over 30 s, while in a rested position. Images were acquired over a 20 min scan, in 5 min acquisitions, beginning 50 min post injection. Brain Aβ load was calculated, using CapAIBL^21^, as the mean standard uptake value ratio (SUVR) of the neocortical region, including the frontal, superior parietal, lateral temporal, lateral occipital, and anterior and posterior cingulate regions normalised to the cerebellar cortex. A cut-off score of 1.35 SUVR was used to categorise participants with low brain Aβ load (Aβ−, SUVR < 1.35) and high brain Aβ load (Aβ+, SUVR ≥ 1.35)^16^.

### Blood collection, measurement of plasma GFAP, plasma Aβ and NF-L, and APOE genotyping

All study participants fasted for a minimum of 10 h overnight prior to blood withdraw employing standard serological methods and processing^16^. Following blood sample processing, plasma fractions were stored at −80 °C until further testing^16^. Plasma GFAP concentrations were measured at Amsterdam University Medical Centers using the Simoa™ GFAP Discovery Kit on the ultra-sensitive Single molecule array (Simoa) platform (HDx instrument) according to the manufacturer’s instructions (catalogue number 102336, Quanterix, Massachusetts, USA). Briefly, plasma samples were added into the aspiration plate of the instrument and diluted four times using assay diluent. Samples were then incubated simultaneously with the capture beads and biotinylated conjugate for 35 min 15 s followed by a wash step and incubation of streptavidin-ß-galactosidase (SBG) for 5 min 15 s. Following a next wash step, the beads were resuspended in a resorufin ß-D-galactopyranoside (RGP) substrate solution for signal generation. GFAP concentrations were calculated using a 4PL 1/Y2 weighted curve fit on the basis of seven calibrator points (excluding the blank value) between 1.37 and 1000 pg/mL, according to the manufacturer’s instructions. The calibrator points were prepared by serial dilution using a stock of concentrated calibrator included in the test kit. Three serum pools spiked with CSF, to obtain three levels (high–medium–low), served as QC samples with average GFAP concentrations of respectively 283.0, 61.0, and 13.6 pg/mL. The repeatability and reproducibility over the three control samples over the two test runs ranged respectively between 0–14 (%CV) and 8–16 (%CV).

Plasma Aβ concentrations were measured employing the Amyblood test that was developed at Amsterdam University Medical Centers in collaboration with ADx NeuroSciences (Ghent, Belgium), on the Simoa platform (HDx instrument, Quanterix), using monoclonal antibodies provided by ADx NeuroSciences^22^. For Aβ1–40, C-terminal-specific ADx103 (2G3, Aβx–40) was used as the capture antibody and N-terminal specific ADx101 (3D6, Aβ1–x) was used as the detector antibody^23^. For Aβ1–42, C-terminal-specific ADx102 (21F12, x–42) was used as the capture antibody and N-terminal-specific ADx101 (3D6) was used as the detector antibody^23^. Briefly, plasma samples were prediluted 20 times for Aβ1–40 and four times for Aβ1–42 into a 96-well polypropylene pre-dilution plate, using assay diluent. Samples were incubated simultaneously with the capture beads and biotinylated conjugate for 60 min followed by a wash step and incubation of SBG for 5 min 15 s. Following a next wash step, the beads were resuspended in a RGP substrate solution for signal generation. For both Aβ1–40 and Aβ1–42, seven non-blank calibrator points in ready to use format (in assay diluent) were used between 1 and 20 pg/mL. The analyte concentrations were calculated using a 4PL non-weighted curve fit. Three non-spiked individual EDTA plasma samples served as QC samples for both Aβ1–40 and Aβ1–42 assays, with an average concentration of respectively 60.7, 112.8, and 74.4 pg/mL for Aβ1–40, and 16.9, 24.7, and 18.5 pg/mL for Aβ1–42. The repeatability over the three control samples over all duplicate values ranged between 0–4 (%CV) and 0–19 (%CV) for Aβ1–40 and Aβ1–42, respectively. The reproducibility over the two independent test runs ranged between 3–7 (%CV) and 0–16 (%CV) for Aβ1–40 and Aβ1–42, respectively.

Plasma NF-L concentrations were also measured using the Simoa platform, as described previously^24^, using two non-competing monoclonal antibodies wherein capture antibody 47:3 and detector antibody 2:1 were used (Uman Diagnostics, Sweden). Apolipoprotein E (*APOE)* genotype was determined from purified genomic DNA extracted from 0.5 mL whole blood, as previously described^16^.

### Statistical analyses

Descriptive statistics including means and standard deviations were calculated for Aβ− and Aβ+ groups, with comparisons employing Student’s *t* tests or Chi-square tests as appropriate. Linear models were employed to compare continuous variables between Aβ− and Aβ+ groups corrected for covariates age, sex, and *APOE* ε4 carrier status. Dependent variables were natural log transformed to better approximate normality and variance homogeneity as required. Spearman’s correlation coefficient (*r*_s_) was employed to investigate correlations between continuous parameters. Logistic regression with Aβ−/+ as response was used to evaluate predictive models and receiver operating characteristic (ROC) curves constructed from the logistic scores. All analyses were carried out using IBM^®^ SPSS^®^ Version 23 and ROC curves were generated using the package Deducer on R (version 3.2.5).

## Results

### Cohort characteristics

Study participant characteristics are presented in Table 1. No significant differences were observed in sex, age, body mass index (BMI), MMSE scores, and the number of SMCs between Aβ− and Aβ + participants. However, the *APOE* ε4 carriage frequency was significantly higher in the Aβ + group compared to the Aβ− group, as expected^25^ (Table 1).

**Table 1:** Cohort characteristics.

	Aβ−	Aβ+	*p*
Sex (male/female)	18/45	13/20	0.281
Age (years, mean ± SD)	77.41 ± 5.45	79.64 ± 5.20	0.057
BMI (mean ± SD)	27.28 ± 4.51	27.36 ± 3.79	0.927
*APOE ε4* carriers (*N* (%))	5 (7.94)	14 (42.42)	<0.0001
MMSE (mean ± SD)	28.52 ± 1.16	28.82 ± 1.07	0.230
Subjective memory complainers (*N* (%))	49 (77.8)	25 (75.76)	0.823
FBB-PET SUVR (mean ± SD)	1.16 ± 0.09	1.71 ± 0.26	—

Baseline characteristics including sex, age, body mass index (BMI), *APOE ε4* status, Mini-mental State Examination (MMSE) scores, subjective memory complainer status (assessed by the Memory Assessment Clinic-Questionnaire (MAC-Q) score), and brain Aβ load represented by the standard uptake value ratio (SUVR) of ligand ^18^F-florbetaben (FBB) in the neocortical region normalised with that in the cerebellum, have been compared between Aβ− (SUVR < 1.35, n = 63) and Aβ+ (SUVR ≥ 1.35, *n* = 33) study participants. Chi-square tests or linear models were employed as appropriate.

### Comparison of plasma GFAP between Aβ− participants and Aβ***+*** participants

**Fig. 1:**
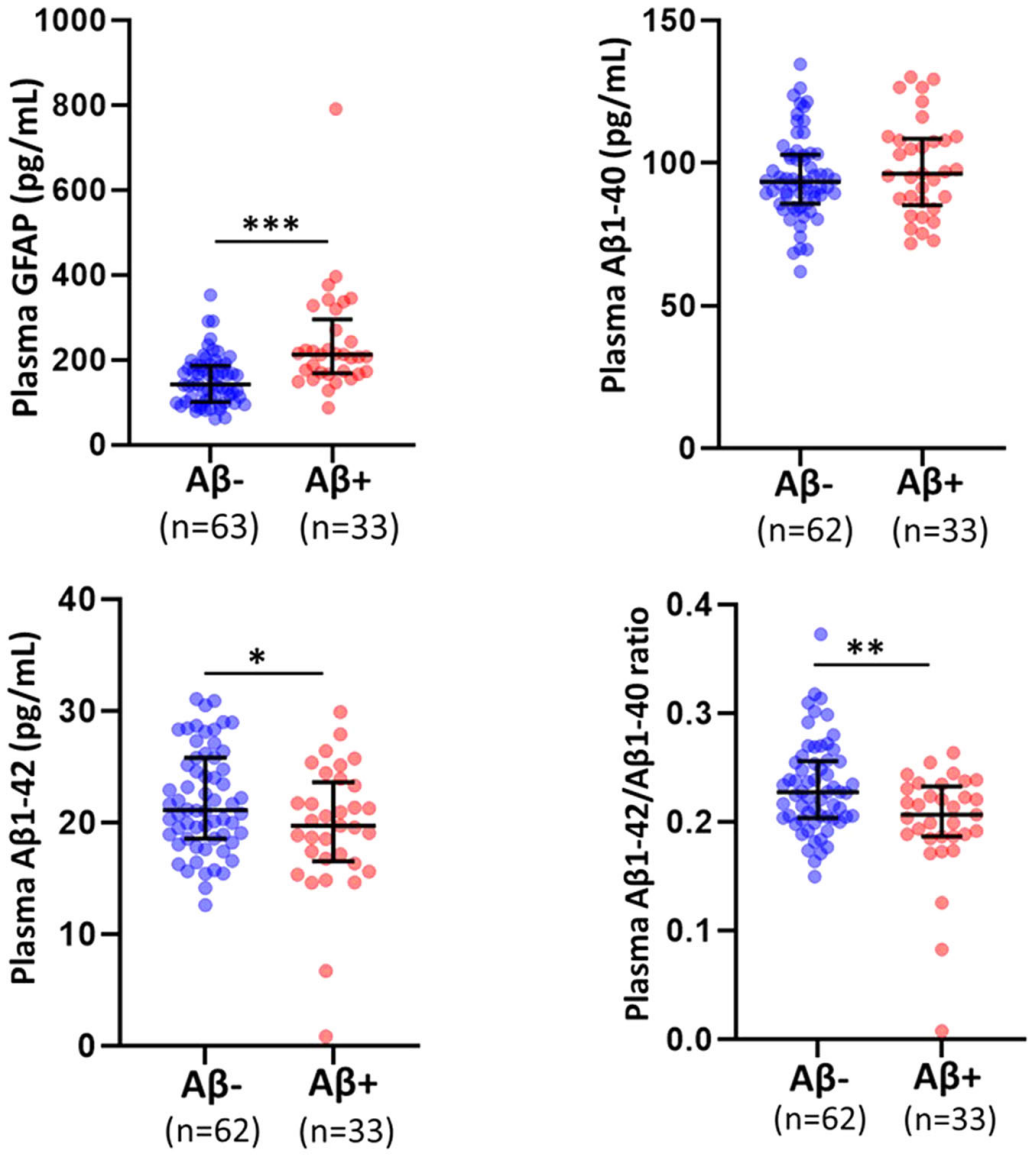
Comparison of plasma GFAP, Aβ1–40, Aβ1–42, and Aβ1–42/Aβ1–40 ratios between Aβ− and Aβ+ cognitively normal older adults. Plasma GFAP, Aβ1–40 and Aβ1–42 levels, and plasma Aβ1–42/Aβ1–40 ratios were compared between cognitively normal older adults with low brain Aβ load (Aβ−) and high brain Aβ load (Aβ+) using linear models. Plasma GFAP concentrations were significantly higher, and plasma Aβ1–42/Aβ1–40 ratios were significantly lower in Aβ+ participants compared to Aβ− participants. The line segment within each jitter plot represents the median of the data and error bars in the graphs represent the interquartile range for the Aβ− and Aβ+ groups. *p < 0.05, **p ≤ 0.001, ***p ≤ 0.0001.

Plasma GFAP concentrations were significantly higher in the Aβ+ group (*n* = 33) compared to the Aβ− group (*n* = 63), before and after adjusting for potential risk factors, age, sex, and *APOE* ε4 status (Fig. 1 and Table 2, *p* < 0.0001).

**Table 2:** Comparison of plasma GFAP between Aβ− and Aβ+ participants.

	Aβ−	(95% CI)	Aβ+	(95% CI)	*p*	*p*^a^
All participants	*n* = 63		*n* = 33			
	151.42 ± 58.49	(129.66–173.18)	240.12 ± 124.88	(210.05–270.18)	**7E−6**	**5.76E−7**
SMC	*n* = 49		*n* = 25			
	152.73 ± 58.18	(126.35–179.11)	252.22 ± 137.75	(215.29–289.16)	^b^**9E−6**	**4.8E−5**
Non-SMC	*n* = 14		*n* = 8			
	146.83 ± 61.58	(112.06–181.60)	202.28 ± 63.81	(156.28–248.27)	0.059	**0.020**

Plasma glial fibrillary acidic protein (GFAP) levels were compared between cognitively normal older adults with low brain Aβ load (Aβ−) and high brain Aβ load (Aβ+) using linear models. All participants were further categorised into subjective memory complainers (SMC, *n* = 74) and non-complainers (non-SMC, *n* = 22). Data are presented in mean ± SD in pg/mL. *p* values in bold font were considered as significant (*p* < 0.05).

^a^*p* represents *p* values adjusted for age, sex, and APOE ε4 status.

^b^Represents *p* values obtained from natural log transformed GFAP concentrations to better approximate normality when required.

On stratifying study participants based on subjective memory complaints (SMC: *n* = 74 and non-SMC: *n* = 22), plasma GFAP continued to remain significantly higher in the Aβ+ SMCs (*n* = 25) compared to Aβ− SMCs (*n* = 49) before and after adjusting for covariates age, sex, and *APOE* ε4 status (Table 2, *p* < 0.0001). In the non-SMCs, plasma GFAP was observed to be significantly higher in Aβ+ non-SMCs (*n* = 8) compared to the Aβ− non-SMCs (*n* = 14) after adjusting for the aforementioned covariates (Table 2, *p* < 0.05).

On stratifying study participants by *APOE* ε4 carriage (ε4 non-carriers: *n* = 77 and ε4 carriers: *n* = 19), significantly higher plasma GFAP concentrations were observed in the Aβ+ group (*n* = 19) compared to the Aβ− group (*n* = 58) within the *APOE* ε4 non-carriers, before and after adjusting for potential risk factors, age, and sex (Supplementary Table 1, *p* < 0.0001). Within the *APOE* ε4 carriers, no significant difference in GFAP concentration was observed between the Aβ+ group (*n* = 14) compared to the Aβ− group (*n* = 5; Supplementary Table 1). This observation could be attributed to the modest sample size of the ε4 carrier subset available within the current study.

### Comparison of plasma Aβ1–40, Aβ1–42, and Aβ1–42/Aβ1–40 ratios between Aβ− participants and Aβ***+*** participants

Plasma Aβ1–40 and Aβ1–42 concentrations and plasma Aβ1–42/Aβ1–40 ratios, measured in the study participants have been presented in Table 3. While no significant differences were observed in plasma Aβ1–40 concentrations between the Aβ− (*n* = 62) and Aβ+ groups (*n* = 33), significant differences in plasma Aβ1–42 concentrations and Aβ1–42/Aβ1–40 ratios were observed between the two groups, wherein plasma Aβ1–42 concentrations and Aβ1–42/Aβ1–40 ratios were lower in the Aβ+ group compared to the Aβ− group before and after correcting for covariates age, sex, and *APOE* ε4 status (Fig. 1 and Table 3, *p* < 0.05).

**Table 3:** Comparison of plasma Aβ1–40, Aβ1–42, and Aβ1–42/Aβ1–40 ratios between Aβ− and Aβ+ participants.

	Aβ−	(95% CI)	Aβ+	(95% CI)	*p*	*p*^a^
All participants	*n* = 62		*n* = 33			
Aβ1–40	95.19 ± 14.78	(91.27–99.10)	98.37 ± 16.88	(93.00–103.74)	0.344	0.659
Aβ1–42	21.96 ± 4.58	(20.70–23.22)	19.54 ± 5.74	(17.81–21.27)	**0.027**	**0.022**
Aβ1–42/Aβ1–40 ratio	0.232 ± 0.042	(0.221–0.243)	0.200 ± 0.050	(0.184–0.215)	**0.001**	**0.004**
SMC	*n* = 50		*n* = 25			
Aβ1–40	95.62 ± 14.74	(91.19–100.05)	98.67 ± 17.54	(92.41–104.94)	0.430	0.624
Aβ1–42	22.05 ± 4.69	(20.58–23.52)	19.12 ± 6.17	(17.02–21.19)	**0.024**	**0.015**
Aβ1–42/Aβ1–40 ratio	0.232 ± 0.044	(0.218–0.245)	0.195 ± 0.056	(0.176–0.214)	**0.003**	**0.003**
Non-SMC	*n* = 12		*n* = 8			
Aβ1–40	93.39 ± 15.48	(83.96–102.83)	97.41 ± 15.68	(85.86–108.97)	0.578	0.093
Aβ1–42	21.57 ± 4.26	(19.02–24.12)	20.89 ± 4.11	(17.77–24.01)	0.727	0.634
Aβ1–42/Aβ1–40 ratio	0.232 ± 0.039	(0.213–0.252)	0.214 ± 0.017	(0.190–0.238)	0.230	0.244

Plasma Aβ1–40 and Aβ1–42 concentrations measured using the Amyblood test (ADx Neurosciences), and their ratio (Aβ1–42/Aβ1–40) were compared between cognitively normal individuals with low brain Aβ load (Aβ−) and high brain Aβ load (Aβ+) using linear models. All participants were further categorised into subjective memory complainers (SMC, *n* = 75) and non-SMC (*n* = 20). Data are presented in mean ± SD in pg/mL. *p* values in bold font were considered significant (*p* < 0.05).

^a^*p* represents *p* values adjusted for age, sex, and APOE ε4 status.

On stratifying study participants based on subjective memory complaints (SMC: *n* = 75 and non-SMC: *n* = 20), plasma Aβ1–42 concentrations, and Aβ1–42/Aβ1–40 ratios continued to remain significantly lower in the Aβ+ SMCs (*n* = 25) compared to Aβ− SMCs (*n* = 50) before and after correcting for covariates age, sex, and *APOE* ε4 status (Table 3, *p* < 0.05). However, no significant difference was observed in plasma Aβ1–42 concentrations, and Aβ1–42/Aβ1–40 ratios between Aβ+ non-SMCs (*n* = 8) and Aβ− non-SMCs (*n* = 12; Table 3).

On stratifying study participants by *APOE* ε4 carriage (ε4 non-carriers: *n* = 76 and ε4 carriers: *n* = 19), significantly lower plasma Aβ1–42 concentrations and Aβ1–42/Aβ1–40 ratios were observed in the Aβ+ group (*n* = 19) compared to the Aβ− group (*n* = 57), within the *APOE* ε4 non-carrier group, after adjusting for potential risk factors, age, and sex (Supplementary Table 2, p < 0.05). Within the *APOE* ε4 carrier group, no significant differences in plasma Aβ1–42 concentrations and Aβ1–42/Aβ1–40 ratios were observed between the Aβ+ group (*n* = 14) compared to the Aβ− group (*n* = 5; Supplementary Table 2). This observation could be attributed to the modest sample size of the ε4 carrier subset available within the current study.

### Evaluation of plasma GFAP and Aβ1–42/Aβ1–40 ratios as predictors of brain Aβ status

**Fig. 2:**
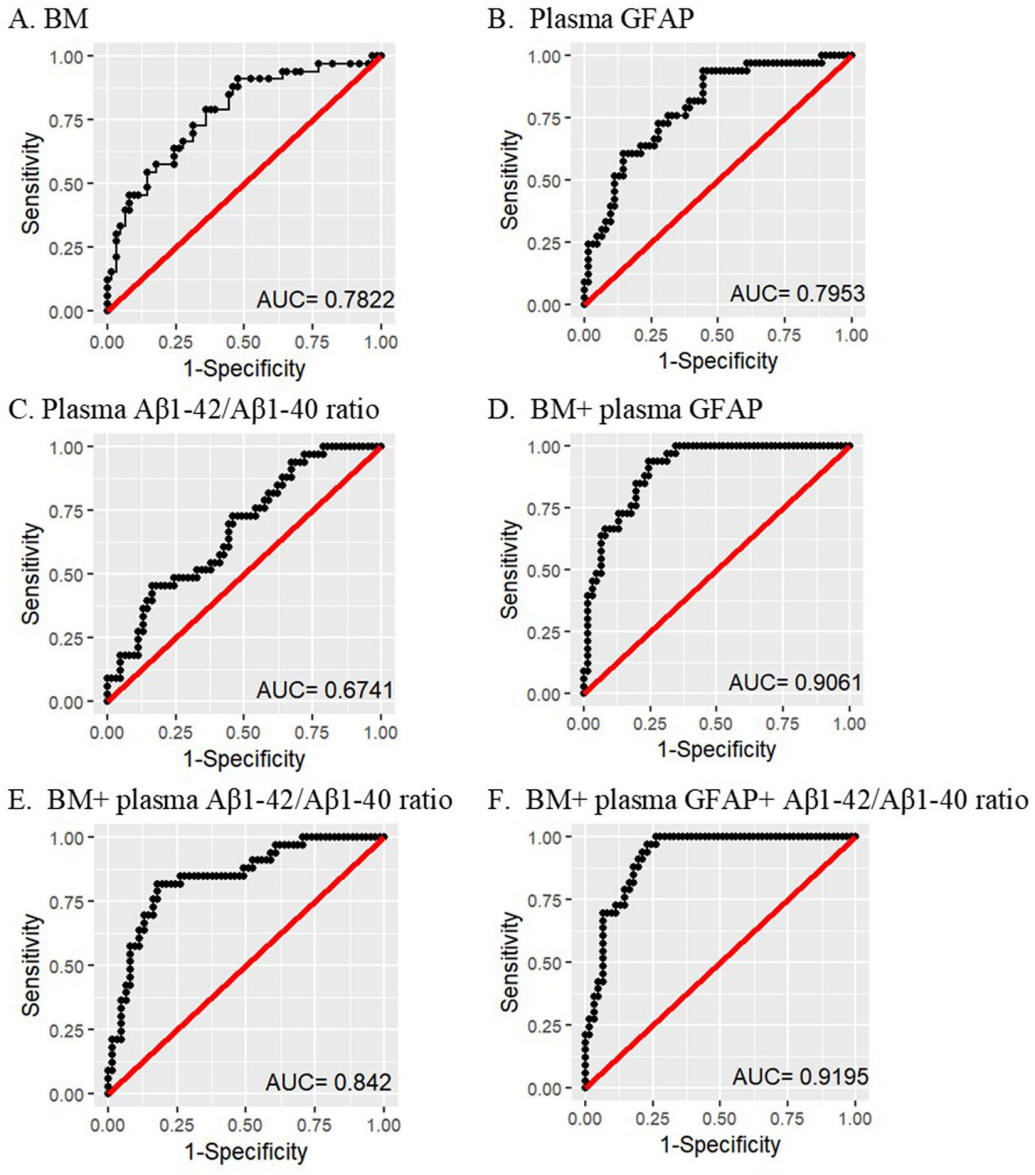
Receiver operating characteristic curves for the prediction of Aβ+ versus Aβ− participants. Receiver operating characteristic (ROC) curves are presented for **A** the ‘base’ model (BM) comprising major risk factors age, sex, and *APOE* ε4 allele status (CI = 0.684–0.880), **B** plasma GFAP (CI = 0.703–0.888), **C** plasma Aβ1–42/Aβ1–40 ratio (CI = 0.563–0.785), **D** BM + GFAP (CI = 0.849–0.964), **E** BM + plasma Aβ1–42/Aβ1–40 ratio (CI = 0.758–0.926), and **F** BM + GFAP + plasma Aβ1–42/Aβ1–40 ratio (CI = 0.867–0.972). The base model was outperformed by models **B**, **D**–**F**. Logistic regression models were employed to perform the analyses. Data from 94 participants were utilised for the analyses (Aβ−, *n* = 61; Aβ+, *n* = 33). GFAP glial fibrillary acidic protein, AUC area under the curve, CI confidence interval.

Plasma GFAP and Aβ1–42/Aβ1–40 ratios were evaluated as potential markers for differentiating between Aβ+ and Aβ− participants, using logistic regression with Aβ+ or Aβ− as response. A ‘base’ model incorporating the major risk factors for AD, namely age, sex, and *APOE* ε4 allele status was generated, and was observed to have an area under the ROC curve (AUC (confidence interval, CI)) of 0.782 (CI = 0.684–0.880) and was outperformed by GFAP alone (AUC = 0.795, CI = 0.703–0.888, sensitivity = 73%, specificity = 72%, *p*(GFAP) = 0.0001), base + GFAP (AUC = 0.906, CI = 0.849–0.964, sensitivity = 85%, specificity = 80%, *p*(GFAP) = 0.00006), base + Aβ1–42/Aβ1–40 ratio (AUC = 0.842, CI = 0.758–0.926, sensitivity = 85%, specificity = 74%, *p*(Aβ42/Aβ40 ratio) = 0.013), and base + GFAP + Aβ1–42/Aβ1–40 ratio (AUC = 0.919, CI = 0.867–0.972, sensitivity = 91%, specificity = 80%, *p*(GFAP) = 0.0001, *p*(Aβ1–42/Aβ1–40 ratio) = 0.042) in distinguishing Aβ+ from Aβ− participants (Fig. 2).

### Association of plasma GFAP with AD risk factors, cognitive measures, and neurodegeneration blood marker, NF-L, in all participants

Plasma GFAP levels correlated with age (*r*_s_ = 0.342, *p* = 0.001), however, no significant association was observed with sex (mean ± SD (pg/mL): males = 160.22 ± 50.89; females = 192.25 ± 110.56; *p* = 0.128) and *APOE* ε4 allele carriage (mean ± SD (pg/mL): non-carriers = 181.98 ± 102.44; carriers = 181.62 ± 68.31; *p* = 0.988). Plasma GFAP was observed to have a significant inverse correlation with working memory and executive function (*r*_s_ = −0.230, *p* = 0.024), but not with verbal, visual, and episodic memory (*r*_s_ = −0.131, *p* = 0.204), while an inverse trend towards statistical significance was observed for the global composite score (*r*_s_ = −0.185, *p* = 0.072). Plasma GFAP levels were not observed to be significantly different between SMCs and non-SMCs (mean ± SD (pg/mL): non-SMC = 167 ± 66.71; SMC = 186.34 ± 103.48; *p* = 0.411). Plasma GFAP was also observed to correlate with plasma NF-L (*r*_s_ = 0.441, *p* = 7E−7).

## Discussion

Findings from the current study show that plasma GFAP levels are increased in cognitively normal older adults with high brain Aβ load, indicating that elevated plasma GFAP may serve as an early blood-based biomarker to identify individuals at risk of AD, prior to the manifestation of clinical symptoms. Our observations build on previous reports of elevated plasma GFAP levels in symptomatic early-onset AD and late-onset AD^7,8^. Further, observations from our study also show that GFAP along with the common AD risk factors (age, sex, and *APOE* ε4 carriage), and plasma Aβ1–42/Aβ1–40 ratio (a blood-based biomarker associated with brain Aβ status^13,15^) distinguished between Aβ− and Aβ+ individuals with 90% sensitivity and 80% specificity, wherein GFAP and plasma Aβ1–42/Aβ1–40 ratio were statistically significant additional predictors of brain Aβ load status, over and above the base model.

GFAP is one of the main intermediate filament proteins in astrocytes that has been thought to be involved in (i) fundamental cellular processes, such as cellular motility^26,27^, proliferation^28,29^, and vesicle trafficking^30^, (ii) interactions between astrocytes and neurons^31–33^, (iii) maintenance of the integrity of the blood–brain barrier and central nervous system myelination^34,35^, and (iv) protection after neuronal injury^36,37^. It could be posited that the elevated plasma GFAP levels observed in the Aβ+ individuals within the current study are attributed to a compromised blood–brain barrier along with an upregulation of GFAP, following astrogliosis, resulting in higher blood GFAP levels in Aβ+ individuals. Findings from the current study are consistent with previous reports of higher GFAP expression in AD brain tissue^38^, and the association between Aβ plaques and a neuroinflammatory response, with astroglial activation and increased GFAP expression^5,39–42^. Furthermore, in line with our findings, PET studies using tracer ^11^C-deuterium-L-deprenyl (used for visualisation of activated astrocytes) also suggest that reactive astrocytosis is potentially a prodromal feature in AD development^4,43,44^.

Interestingly, within the current study, plasma GFAP was observed to be significantly higher in the Aβ+ group (compared to the Aβ− group), in both SMCs and non-SMCs after adjusting for potential confounding variables, although the significance level observed in SMCs was to a much greater extent compared to that observed in the non-SMCs. However, plasma Aβ1–42/Aβ1–40 ratios were only significantly lower in Aβ+ participants (compared to the Aβ− participants) within the SMC subset, after stratifying participants based on their SMC status. This observation is consistent with a previous study employing the same cohort using a different Aβ measurement assay^13^.

In addition, the current study also stratified participants based on *APOE* ε4 allele carriage and observed that while GFAP was significantly higher in the Aβ+ *APOE* ε4 non-carriers (compared to the Aβ− *APOE* ε4 non-carriers), and Aβ1–42/Aβ1–40 ratios were significantly lower in the Aβ+ *APOE* ε4 non-carriers (compared to the Aβ− *APOE* ε4 non-carriers), plasma GFAP levels, and Aβ1–42/Aβ1–40 ratios were not significantly altered in Aβ+ versus Aβ− *APOE* ε4 carriers. This observation could be attributed to the modest sample size of the ε4 carrier subset available within the current study. Nonetheless, the observations of plasma GFAP and Aβ1–42/Aβ1–40 ratios remaining significantly altered between Aβ− and Aβ+ participants within the *APOE* ε4 non-carrier subset from this exploratory analysis may be viewed as a beneficial feature for early AD biomarkers, given that the presence of the *APOE* ε4 allele in itself is a major risk factor for the disease.

Within the current study, we observed a correlation between plasma GFAP and age, consistent with a previous report^8^. Increased GFAP expression with age has also been reported in the brain^45,46^, caused by increased GFAP transcription, which has been suggested to be caused due to increased oxidatively damaged protein during ageing^47^. In addition, within the current study, plasma GFAP inversely correlated with cognitive performance, particularly with working memory and executive function; however, further studies are required to validate these observations.

Interestingly, we observed a highly significant correlation between plasma GFAP and plasma NF-L (comparison of plasma NF-L levels between Aβ− and Aβ+ participants are presented in Supplementary Table 3). This correlation observed between plasma GFAP and plasma NF-L, revealing the association between astrocytic damage and axonal damage, is consistent with previous reports^48,49^.

In addition, the presence GFAP-IgG seropositivity in autoimmune GFAP astrocytopathy, an autoimmune disease of the nervous system^50^ and the increased risk of AD in individuals with autoimmune diseases^51^, along with our observations of increased plasma GFAP in individuals at risk of AD, warrant further investigation into the link between AD and autoimmune disorders, and the investigation of possible mechanisms associated with this link.

It is acknowledged that the current study has limitations, given its modest sample size and cross-sectional design, particularly after stratifying the cohort into SMC and non-SMC subsets or *APOE* ε4 non-carrier and carrier subsets. Therefore, further studies are required to validate the current findings in larger independent cohorts, using both cross-sectional and longitudinal study designs. Longitudinal studies will provide more insight into the trajectory of plasma GFAP alterations associated with the progression of AD pathogenesis. In addition, since increased plasma GFAP has been reported to be associated with other dementias^8^ and neurodegenerative disorders^49,52^, the specificity of GFAP as a biomarker to identify cognitively normal older adults at risk of AD warrants further research. However, it must also be noted that while GFAP may be associated with other dementias and neurodegenerative disorders, our data clearly show a significant positive association between GFAP and brain Aβ load measured by PET (Supplementary Fig. 2), a gold standard biomarker for AD.

To conclude, the current study is the first to demonstrate increased plasma GFAP levels in cognitively normal older adults at risk of AD. These observations suggest that astrocytic damage begins from the pre-symptomatic stage of AD and is associated with brain Aβ load. Further, observations from the current study show that the combination of plasma GFAP and plasma Aβ1–42/Aβ1–40 ratios along with the major AD risk factors, have the potential to differentiate between Aβ+ and Aβ− individuals, albeit further studies in independent cohorts are required to validate these findings. The utilisation of plasma GFAP to identify individuals at risk of AD (Aβ+ individuals), decades before the onset of AD clinical symptoms for clinical trials could assist with reducing the considerable screening costs, thereby facilitating much needed prevention programmes and clinical intervention trials.

## Supplementary information

Supplementary Material
