# Supplementary material for: Plasma glial fibrillary acidic protein is elevated in cognitively normal older adults at risk of Alzheimer’s disease
Source: Transl Psychiatry. 2021 Jan 11;11:27. doi: 10.1038/s41398-020-01137-1 (PMC7801513; doi:10.1038/s41398-020-01137-1)
Supplement: Supplementary file 1 — Supplementary Material [file 41398_2020_1137_MOESM1_ESM.docx]

**Supplementary Material**

**Plasma glial fibrillary acidic protein is elevated in cognitively normal older adults at risk of Alzheimer’s disease**

Pratishtha Chatterjee, Ph.D. ^a,b^, Steve Pedrini, M.Sc. ^b^, Erik Stoops, M.Sc. ^c^, Kathryn Goozee, MCN ^a,b,d,e,f,g,^, Victor L. Villemagne, M.D. ^h^, Prita R. Asih, Ph.D. ^a^, Inge M. W. Verberk, Ph.D. ^i^, Preeti Dave, M.Sc. ^a,e^, Kevin Taddei, B.Sc. ^b,j^, Hamid R. Sohrabi, Ph.D. ^a,b,j,k^, Henrik Zetterberg, M.D., Ph.D. ^l,m,n,o^, Kaj Blennow, M.D., Ph.D. ^l,m^, Charlotte E. Teunissen, Ph.D. ^i^, Hugo M. Vanderstichele, Ph.D. ^p^, Ralph N. Martins, Ph.D.^a,b,d,f,g,j,^*

*a: Department of Biomedical Sciences, Macquarie University, North Ryde, NSW, Australia*

*b: School of Medical and Health Sciences, Edith Cowan University, Joondalup, WA, Australia*

*c: ADx NeuroSciences, Gent, Belgium*

*d: KaRa Institute of Neurological Disease, Sydney, Macquarie Park, Australia*

*e: Anglicare, Sydney, Castle Hill, NSW, Australia*

*f: School of Psychiatry and Clinical Neurosciences, University of Western Australia, Crawley, WA, Australia*

*g: The Cooperative Research Centre for Mental Health, Carlton South, Australia*

*h: Department of Molecular Imaging & Therapy, Austin Health, Melbourne, VIC, Australia*

*i: Neurochemistry Laboratory, Department of Clinical Chemistry, Amsterdam Neuroscience, Amsterdam University Medical Centers, Amsterdam, Netherlands.*

*j: Australian Alzheimer’s Research Foundation, Nedlands, WA, Australia*

*k: Centre for Healthy Ageing, School of Psychology and Exercise Science, College of Science, Health, Engineering and Education, Murdoch University, Murdoch, WA, Australia*

*l: Department of Psychiatry and Neurochemistry, Institute of Neuroscience and Physiology, University of Gothenburg, Mölndal, Sweden*

*m: Clinical Neurochemistry Laboratory, Sahlgrenska University Hospital, Mölndal, Sweden*

*n: Department of Neurodegenerative Disease, UCL Institute of Neurology, Queen Square, London, United Kingdom*

*o: UK Dementia Research Institute at UCL, London, United Kingdom*

*p: Biomarkable, Gent, Belgium*

**Supplementary Table 1. Comparison of plasma** **GFAP concentrations between Aβ- and Aβ+ participants after stratification by *APOE* ε4 carriage.** Plasma glial fibrillary acidic protein (GFAP) levels were compared between cognitively normal older adults with low brain Aβ load (Aβ-) and high brain Aβ load (Aβ+) using linear models, after stratifying participants into *APOE* ε4 non-carriers (n=77) and carriers (n=19). † represents p-values obtained from natural log transformed GFAP concentrations. p^a^ represents p-values adjusted for age and sex. Data are presented in mean±SD in pg/mL.

|  | **Aβ -** | **(95% CI)** | **Aβ +** | **(95% CI)** | **p** | **p^a^** |
| --- | --- | --- | --- | --- | --- | --- |
| ***APOE* ε4 non-carriers** | **n=58** |  | **n=19** |  |  |  |
|  | 151.17±56.84 | (128.28-174.07) | 276.02±147.26 | (236.02-316.02) | †**2.3E-7** | †**7.05E-7** |
| ***APOE* ε4 carriers** | **n=5** |  | **n=14** |  |  |  |
|  | 154.26±83.55 | (89.97-218.55) | 191.39±62.63 | (152.97-229.81) | .310 | .107 |

**Supplementary Table 2. Comparison of plasma Aβ40, Aβ42 and Aβ42/Aβ40 ratios between Aβ- and Aβ+ participants after stratification by *APOE* ε4 carriage.** Plasma Aβ40 and Aβ42 concentrations measured using the Amyblood test (ADx Neurosciences) and their ratio (Aβ42/Aβ40) were compared between cognitively normal older adults with low brain Aβ load (Aβ-) and high brain Aβ load (Aβ+) using linear models, after stratifying participants into *APOE* ε4 non-carriers (n=76) and carriers (n=19). p^a^ represents p-values adjusted for age and sex. Data are presented in mean±SD in pg/mL.

|  | **Aβ -** | **(95% CI)** | **Aβ +** | **(95% CI)** | **p** | **p^a^** |
| --- | --- | --- | --- | --- | --- | --- |
| ***APOE* ε4 non-carriers** | **n=57** |  | **n=19** |  |  |  |
| **Aβ40** | 94.82±15 | (90.65-98.99) | 103.12±18.05 | (95.90-110.34) | .051 | .304 |
| **Aβ42** | 22.02±4.57 | (20.68-23.35) | 19.86±6.36 | (17.55-22.18) | .113 | **.038** |
| **Aβ42/Aβ40 ratio** | 0.23±0.04 | (0.22-0.24) | 0.19±0.05 | (0.17-0.21) | **.001** | **.002** |
| ***APOE* ε4 carriers** | **n=5** |  | **n=14** |  |  |  |
| **Aβ40** | 99.33±12.52 | (87.08-111.59) | 91.92±13.12 | (84.60-99.24) | .288 | .397 |
| **Aβ42** | 21.29±5.23 | (16.55-26.02) | 19.10±4.95 | (16.27-21.93) | .414 | .447 |
| **Aβ42/Aβ40 ratio** | 0.22±0.055 | (0.17-0.26) | 0.21±0.05 | (0.18-0.24) | .803 | .637 |

**Supplementary Table 3. Comparison of plasma NF-L between Aβ- and Aβ+ participants.**

Plasma NF-L concentrations compared between cognitively normal older adults with low brain Aβ load (Aβ-) and high brain Aβ load (Aβ+) using linear models, are presented in all participants and after stratifying by *APOE* ε4 carrier status. p^a^ represents p-values adjusted for age and sex. Data are presented in mean±SD in pg/mL.

|  | **Aβ -** | **(95% CI)** | **Aβ +** | **(95% CI)** | **p** | **p^a^** |
| --- | --- | --- | --- | --- | --- | --- |
| **All participants** | **n=63** |  | **n=33** |  |  |  |
|  | 34.73±16.46 | (30.41-39.06) | 39.37±18.81 | (33.40-45.35) | .215 | .447 |
| ***APOE* ε4 non-carriers** | **n=58** |  | **n=19** |  |  |  |
|  | 34.75±16.51 | (30.21-39.29) | 44.43±19.80 | (36.50-52.36) | **.038** | .299 |
| ***APOE* ε4 carriers** | **n=5** |  | **n=14** |  |  |  |
|  | 34.50±17.72 | (19.36-49.63) | 32.51±15.49 | (23.47-41.56) | .815 | .980 |

**Supplementary Figure 1: Flowchart illustrating participants included within the current study.**

**
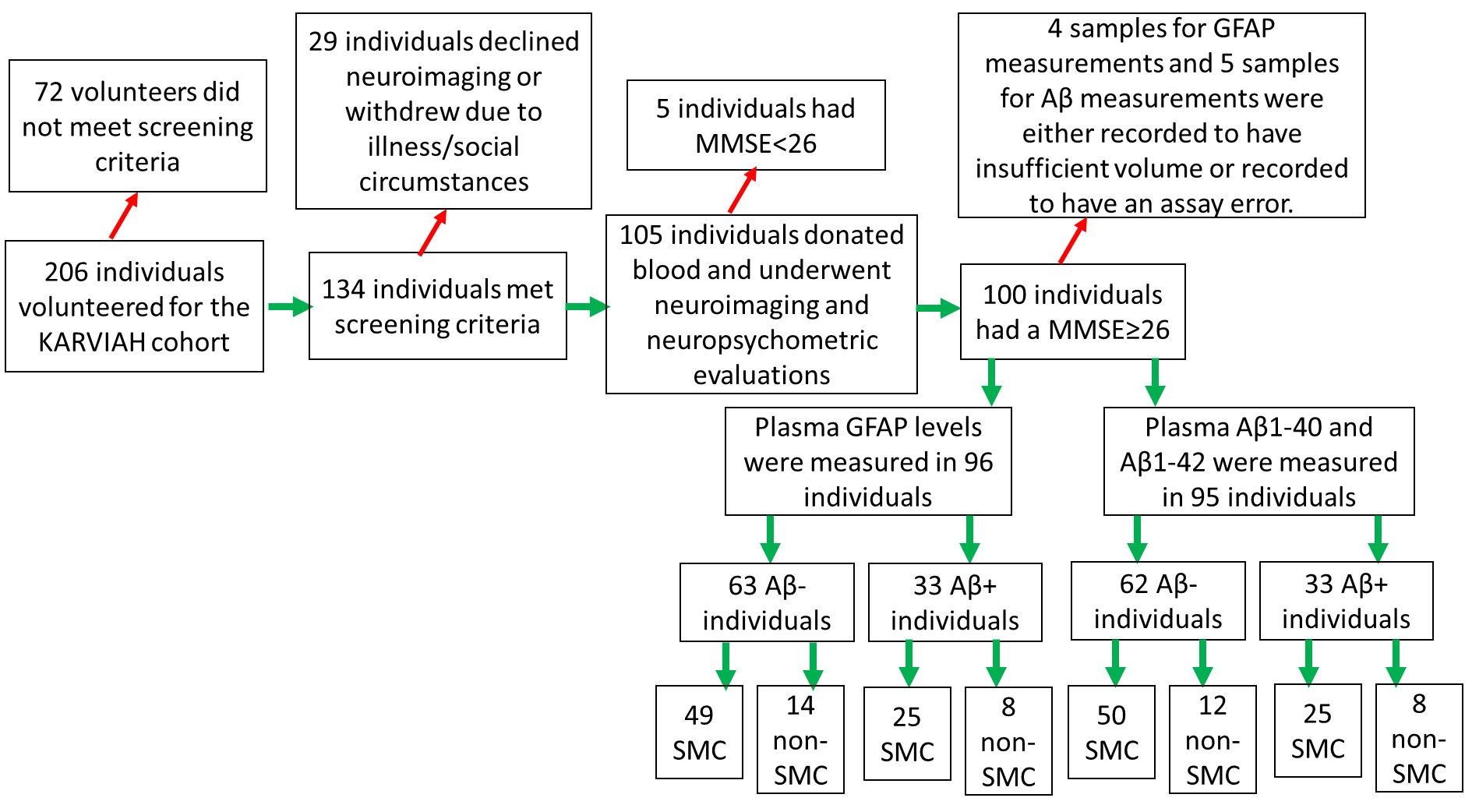
**

**Supplementary Figure 2: Association between plasma GFAP and brain Aβ load measured using PET.**

**
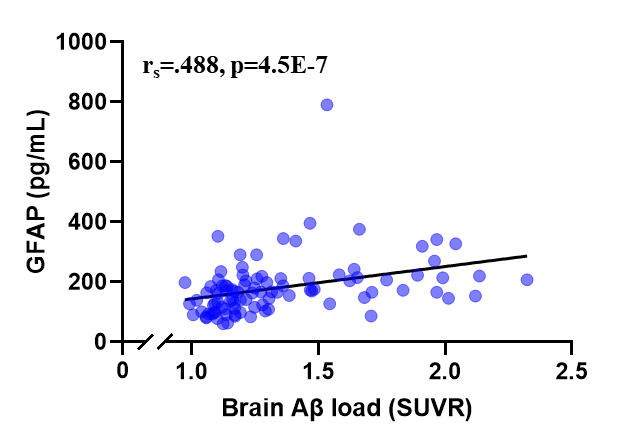
**
